# Eysenck's Personality Questionnaire Scores of Heroin Addicts in Pakistan

**DOI:** 10.7759/cureus.65156

**Published:** 2024-07-22

**Authors:** Mohammad Ali, Urbah Viqar

**Affiliations:** 1 Internal Medicine, Dr Akbar Niazi Teaching Hospital, Islamabad, PAK; 2 Psychiatry, Arch Hillingdon, London, GBR

**Keywords:** psychoticism, extraversion, neuroticism, heroin addicts, eysenck's personality questionnaire

## Abstract

Objective

The main objective of the study was to find Eysenck's Personality Questionnaire scores of heroin addicts in Pakistan and to compare the EPQ scores with demographic variables such as age, gender, and duration of addiction.

Methodology of the study

This cross-sectional study was conducted in Islamabad, Pakistan. Data was collected from 300 participants. The participants were recruited from various drug rehabilitation centers and clinics across Pakistan. Patients diagnosed with heroin addiction by a qualified medical professional and currently undergoing treatment or rehabilitation for heroin addiction were included in the study. Data was collected through a purposive sampling technique.

Results

Data was collected from 300 participants, including both males and females. The age range included subjects from 18 years to 60 years. Heroin addicts in Pakistan have a mean extraversion score of 12.4 (SD=4.8), with scores ranging from 5 to 20, indicating average levels of sociability. Neuroticism had a mean score of 17.6 (SD=5.2), with a range from 7 to 25, suggesting high emotional instability and anxiety. Psychoticism had a mean score of 8.3 (SD=3.9), with scores between 2 and 15, indicating moderate levels of aggressive and antisocial behavior.

Conclusion

It is concluded that heroin addicts in Pakistan exhibit high levels of neuroticism, moderate levels of extraversion, and psychoticism. Younger addicts and those with a shorter duration of addiction display higher levels of these traits, indicating a need for personalized and targeted intervention strategies.

## Introduction

Understanding the psychological profiles of individuals struggling with substance abuse is crucial for developing effective treatment and rehabilitation programs. One of the critical tools used to assess personality traits in various populations is the Eysenck's Personality Questionnaire (EPQ), which measures three significant dimensions of personality: extraversion (E), neuroticism (N), and psychoticism (P) based on Eysenck's personality theory [1]. Herein, opioid dependence has overall transformed into one of the most critical public health concerns in the context of Pakistan with worrisome socio-economic consequences. This study seeks to assess the personality profile of heroin addicts using EPQ to find out the psychological disposition of intending heroin addicts [2].

Pakistan's situation has observed a general inclination to enhance cases of drug abuse; particularly, the youth have been highlighted as the most numerous in this menace, and many people have conducted research in this area in order to gain an understanding of the issue [3]. One such area of research interest that has only started to develop and that has offered me research questions to approaches to personality has been that of drug abuse [4].

An increased number of studies have pointed out the fact that specific personality characteristics, including impulsivity, neuroticism, aggressiveness as well as extraversion, are exhibited by drug users [5]. Thus, the study of personality in addiction is multifaceted, and features may change with the variety of options, like the type of drugs used, frequency, and intensity [5]. In investigating the personality traits in drug addicts, moreover, it was identified that one attribute was related to them: impulse control is connected with some personality characteristics [6]. With the population of drug users on the increase in Pakistan, there are 6.7 million drug users in Pakistan, and among them, 78% of the clients were male, and 22% were female clients. Such natural and synthetic medicines were reported to be used by eight percent of Pakistan's population between the ages of 15 and 64 [7].

The population of addicted persons using heroin is estimated to be 8 million, while that of opium addict persons is three million, and four million people are still involved in cannabis usage. Another popular trend is the use of ice among privileged youth and students [8]. Several reports were also published to elaborate on the possible consequences of drug intake, which may cause contaminated sleep, hallucinations, fatigue, loss of interest in enjoyable activities, suicidal tendencies, tachycardia, hypertension, and ataxia. There is a decrease in psychological suffering when an upbeat personality is exercised under challenging situations, thus improving psychological health [9].

As research argues, some personality factors correlate with self-reported psychological distress. One could conclude that a high level of neuroticism and a low level of agreeableness, consciousness, openness to experience, and extraversion are positive indicators. Heroin addiction is not only a medical condition but also a complex psychosocial problem that affects individuals' behavior, emotions, and social interactions [10]. Personality traits, which are relatively stable characteristics influencing how individuals think, feel, and behave, play a pivotal role in understanding addiction. The EPQ, developed by Hans Eysenck, is a well-established instrument that has been extensively used in psychological research to categorize personality traits into three primary dimensions: extraversion (sociability and liveliness), neuroticism (emotional instability and anxiety), and psychoticism (aggressiveness and antisocial behavior) [11]. Thus, sociocultural factors, such as peer influence, social networks as well as employment status, play a major role in heroin addiction. In terms of economic factors, poverty plays a major role in addiction. In regards to accessibility of the drug in Pakistan, the proximity to the Afghanistan-Pakistan border is a major heroin trafficking route, and the ease of access to heroin through social networks and dealers makes it easier to procure heroin.

However, a literature review reveals the absence of a significant number of empirical studies concerning the psychological aspect of addiction in this region [12]. Thus, the present study aims to investigate the specific personality questionnaire index among the researched sample of heroin addicts in order to contribute to further studies in the field and, more specifically, to develop intervention strategies [13].

## Materials and methods

This cross-sectional study was conducted in Islamabad, Pakistan, over a period of one year, starting from May 2023 to May 2024. The aim of the study is to investigate the effects of heroin addiction on individuals undergoing treatment or rehabilitation. A total of 300 participants were recruited from various drug rehabilitation centers and clinics (both government and private) across Pakistan. The sample size was calculated by the WHO sample size calculator. The age range included subjects from 18 years to 60 years.

The participants were selected based on specific criteria, including a diagnosis of heroin addiction by a qualified medical professional and current enrollment in a treatment or rehabilitation program for heroin addiction. Subjects who were less than 16 years old or more than 60 years of age, HIV positive, with active tuberculosis or hepatitis, or those who were using substances different from heroin, such as cocaine, methamphetamine, and marijuana, were excluded from the study.

The data collection process involved a purposive sampling technique, as it allowed us to select participants who fit the criteria. Rehabilitation centers and clinics were approached to identify potential participants who fit the study requirements. This technique allowed the researchers to target a specific population and gather data that was rich in information and insight. Data collection was conducted through self-administered questionnaires. Permission to carry out the research project was granted by the institutional review board of Islamabad Medical and Dental College. The Eysenck Personality Questionnaire (EPQ) was used as the main instrument designed to assess participants' personalities due to its established reliability and validity in measuring different personality dimensions (extraversion, neuroticism, and psychoticism), which is relevant to understanding heroin addiction. This questionnaire consists of a series of statements to which the participants respond with "Yes" or "No," assessing three major personality dimensions. Another model of personality inventory is Eysenck's Personality Questionnaire, which includes extraversion (E), neuroticism (N), and psychoticism (P). Professional and well-trained research assistants were used to complete the EPQ administration to minimize variation and errors. This was done to enhance the anonymity of the data collected; the participants were numbered, and all the responses were obtained on secure forms and kept. Participants took an average of 10 minutes to complete the questionnaire.

Data was analyzed using SPSS version 29 (IBM Inc., Armonk, New York). The Chi-squared test was used to find out the p-value to see if the correlation between various variables was statistically significant or not. A p-value of <0.05 showed that the EPQ scores between the variables were statistically significant, and a p-value of >0.05 showed that the EPQ scores between the variables were not statistically significant. The descriptive statistics for the EPQ scores were presented in mean and SD.

## Results

Data was collected from 300 participants, including both males and females. The age range was included from 18 years to 60 years. Heroin addicts in Pakistan have a mean extraversion score of 12.4 (SD=4.8) with scores ranging from 5 to 20, indicating average levels of sociability. Neuroticism had a mean score of 17.6 (SD=5.2) with a range from 7 to 25, suggesting high emotional instability and anxiety. Psychoticism had a mean score of 8.3 (SD =3.9), with scores between 2 and 15, indicating moderate levels of aggressive and antisocial behavior (Table 1).

**Table 1:** Descriptive statistics of Eysenck's Personality Questionnaire scores

Personality trait	Mean	Standard deviation	Minimum	Maximum
Extraversion (E)	12.4	4.8	5	20
Neuroticism (N)	17.6	5.2	7	25
Psychoticism (P)	8.3	3.9	2	15

**Figure 1:**
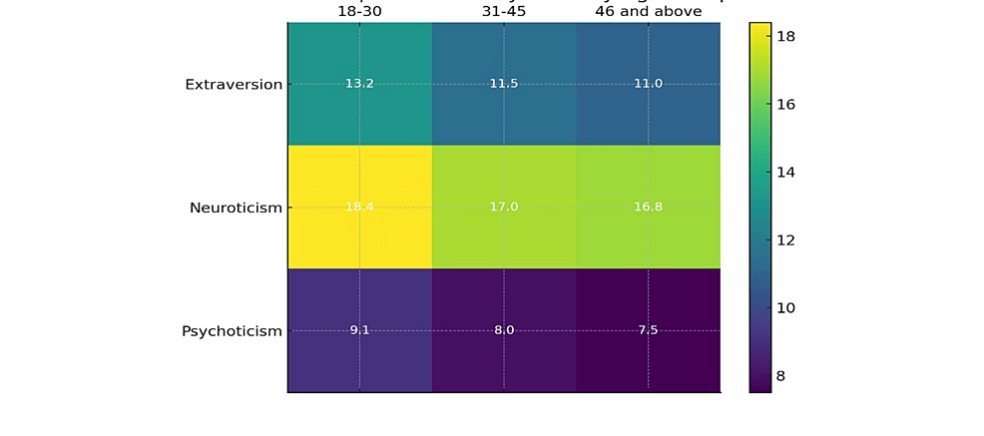
Heatmap of personality traits by age group

For extraversion, the 18-30 age group had the highest mean score of 13.2, followed by the 31-45 age group at 11.5, and the 46 and above age group at 11.0, with a p-value of 0.02 indicating that the differences in extraversion scores across the age groups (18-30, 31-45, 46 and above) are statistically significant. Neuroticism scores were highest in the 18-30 age group (18.4), followed by the 31-45 age group (17.0) and the 46 and above age group (16.8), with a p-value of 0.03 (statistically significant). Psychoticism scores were also highest in the 18-30 age group (9.1), followed by the 31-45 age group (8.0) and the 46 and above age group (7.5), with a p-value of 0.04 (statistically significant; Table 2). The differences in extraversion, neuroticism, and psychoticism scores across age groups were statistically significant. Figure 1 depicts heatmap of personality traits by age group.

**Table 2:** Inferential statistics of Eysenck's Personality Questionnaire scores by age group

Age group	Extraversion (E)	Neuroticism (N)	Psychoticism (P)
18-30 (n=120)	13.2	18.4	9.1
31-45 (n=100)	11.5	17.0	8.0
46 and above (n=80)	11.0	16.8	7.5
p-value	0.02	0.03	0.04

Participants with less than one year of addiction had the highest mean scores for extraversion (13.5), neuroticism (19.0), and psychoticism (9.5). Those with one to five years of addiction had mean scores of 12.0 for extraversion, 17.8 for neuroticism, and 8.3 for psychoticism. Participants with more than five years of addiction had the lowest mean scores, with 11.2 for extraversion, 16.5 for neuroticism, and 7.8 for psychoticism (Table 3). The p-values are 0.01 for extraversion, 0.02 for neuroticism, and 0.03 for psychoticism. This suggests that the differences in scores for these traits are statistically significant.

**Table 3:** Inferential statistics of Eysenck's Personality Questionnaire scores by duration of addiction

Duration of addiction	Extraversion (E)	Neuroticism (N)	Psychoticism (P)
Less than 1 year (n=50)	13.5	19.0	9.5
1-5 years (n=150)	12.0	17.8	8.3
More than 5 years (n=100)	11.2	16.5	7.8
p-value	0.01	0.02	0.03

Males had a mean extraversion score of 12.5, a neuroticism score of 17.5, and a psychoticism score of 8.4. Females had slightly lower mean scores for extraversion (12.0) and psychoticism (8.0) but a higher neuroticism score (18.0). The p-values for these comparisons were 0.25 for extraversion, 0.32 for neuroticism, and 0.28 for psychoticism, indicating that these differences are not statistically significant (Table 4).

**Table 4:** Inferential statistics of Eysenck's Personality Questionnaire scores by gender

Gender	Extraversion (E)	Neuroticism (N)	Psychoticism (P)
Male (n=250)	12.5	17.5	8.4
Female (n=50)	12.0	18.0	8.0
p-value	0.25	0.32	0.28

## Discussion

The results of this study highlight significant personality traits among heroin addicts in Pakistan. High levels of neuroticism, indicating emotional and anxiety intolerance, may contribute to both the onset and maintenance of heroin addiction. The results suggest medium levels of extraversion and psychoticism, which reveal the subject's tendencies in sociability and antisocial behavior, respectively [14]. This assessment of a sample of heroin addicts reveals the following average profile of the substance-dependent clientele: They are highly neurotic, moderately extroverted, and moderately psychotic. The conclusions drawn from such studies lead to the understanding of the fact that heroin addicts are likely to be emotionally unstable and anxious, which points towards the probability of likely having contributed toward the initiation of drug use and sustenance of the habit [15]. 

The values obtained for extraversion and psychoticism are moderate, which has defined their presence but not as the defining traits throughout all the participants [16]. The statistical analysis showed a clear distinction in the personality in terms of age. Participant age also significantly affected the personality measures; the younger participant group, especially those between 18 and 30 years, had significantly higher mean scores on E, N, and P compared to the older participants [17]. This implies that present-day young heroin-dependent persons may be more socially mobilized but with emotive impressionability and are more inclined to aggressiveness and antisociality. It may be speculated that participants of a younger age could show elevated means on the measures of extraversion, neuroticism, and psychoticism attributable to multiple social and developmental factors. They tend to be more Extraverted and Neurotic by the time they get to be adolescents and early adults because these are the stages where emotions are more volatile, risks are more frequently taken, and social acceptance is more desirable [14]. Further, the brain's frontal lobe, which controls decision-making capacity and impulse control, is not fully developed until the teen years or later; as such, teens may be more prone to impulsive behavior and, in turn, more vulnerable to the gateway effects of drugs. Such characteristics can pose barriers to the management and healing process, noting the categorical need for young people's specific treatment methods that encapsulate the respective psychological disposition of youthful addicts [18]. 

It was also evident that changes depended on the period of addiction condition [18]. There were differences in the scores of personality, namely, extraversion and neuroticism, between the participants with less than one year duration of addiction and those with more than one year duration of addiction [19]. This means that the recent addicts would be comparatively more sociable and emotionally labile. Hence, the scores on psychoticism are higher in this group, implying that people within this group will be more inclined to exhibit aggressive and antisocial behaviors. These outcomes imply the necessity of employing appropriate preventive measures and individual methods of treating new addicts in an attempt to mitigate these increased psychological repercussions.

Contrary to the anticipated results, an analysis of variance did not reveal differences between male and female participants about extraversion, neuroticism, or psychoticism [20]. This implies that gender does not have any influence on the type of personality among the people with a heroin addiction in this sample. This indicates that psychological factors vary depending on the gender and, therefore, it is advisable to design programs for intervention that will suit the needs of both male and female addicts [21]. 

Those who are relatively new to heroin addiction may display elevated rates of extraversion, neuroticism, and psychoticism. The Stress-Diathesis model says that those who test high on neuroticism might use drugs to help deal with stress, and those who test high on extraversion might engage in drugs more, in a more reckless manner, and to socialize. Similarly, in the Personality-Disorder model, there are personality traits that, in connection with high psychoticism, may also be attributed to substance abuse [20]. Essentially, these findings support previous work to suggest that those with these attributes exhibit behaviors associated with addiction. Other possible demographic parameters that could link to gender differences in neuroticism could include societal pressure and possible differences in the way the male and female transcript emotions in a given society.

In light of the high levels of neuroticism reported here, control of emotions and stress should be included in the therapeutic programs for heroin addicts. Psychoeducation and rational-emoting therapies include cognitive-behavioral therapy (CBT), dialectical behavior therapy (DBT), and motivational interviewing. Also, the differences regarding the age and duration of the treatment yielded personality differences, which could implicate the necessity of individualized treatment considering the given parameters [22]. More methods focused on specific psychological requirements and with greater intensity can be appropriate to subject younger addicts and those who have had an addiction for a shorter period. However, it is also essential to consider this study's limitations.

Compared to probability sampling, purposive sampling may result in a less representative sample, and hence, the generalization of the results to the population of heroin addicts may be affected. As for the self-report bias, the overall results can be biased due to such factors as social desirability or distortions in the perception of one's personality [23]. Studying the patient's personality changes over time is critical for gathering meaningful data on how these changes affect their treatment. Following people for a longer time allows the researchers to easily determine if the measured traits are fairly static or variable and how this influences the success of the intervention. This could supply important details concerning the approach that individuality features change with recuperation and how treatment interventions need to be fine-tuned to optimize durability results [23].

## Conclusions

It is concluded that heroin addicts in Pakistan exhibit high levels of neuroticism, moderate levels of extraversion, and psychoticism. Younger addicts and those with a shorter duration of addiction display higher levels of these traits, indicating a need for personalized and targeted intervention strategies, such as cognitive behavioral therapy (CBT) and dialectical behavior therapy (DBT), as well as providing peer support groups. Addressing emotional instability and anxiety through tailored treatment plans, including medication-assisted treatment, is crucial for improving recovery outcomes in this population. Furthermore, our findings highlight the importance of national health policies to prioritize individualized treatment strategies in the process of treating heroin dependency. The new approaches have to address the lack of effectiveness of the old ones by helping policymakers integrate more usable, individualized treatments into addiction treatment practices. It is essential for the Pakistani practitioners and policymakers to introduce these specific treatment modalities and conduct more studies in order to identify effective ways to rehabilitate the clients suffering from heroin addiction and improve their health conditions.
